# Structural heart defects associated with ET_B_ mutation, a cause of Hirschsprung disease

**DOI:** 10.1186/s12872-021-02281-2

**Published:** 2021-10-02

**Authors:** Ko-Chin Chen, Ko-Chien Chen, Zan-Min Song, Geoffrey D. Croaker

**Affiliations:** 1 Australian National University Medical School, Florey Building 54 Mills Road, Acton, ACT 2601 Australia; 2 MD Anderson Cancer Centre, University of Texas, Houston, TX 77030 USA; 3 The John Curtin School of Medical Research, Australian National University Medical School, Acton, ACT 2601 Australia; 4 Paediatric Surgery, The Canberra Hospital, Garran, ACT Australia

**Keywords:** Hirschsprung disease, Heart defects, Endothelin-B mutation, Spotting-lethal rat

## Abstract

**Background:**

HSCR, a colonic neurocristopathy affecting 1/5000 births, is suggested to associate with cardiac septal defects and conotruncal malformations. However, we question subtle cardiac changes maybe more commonly present due to multi-regulations by HSCR candidate genes, in this instance, ET_B_. To investigate, we compared the cardiac morphology and quantitative measurements of *sl/sl* rat to those of the control group.

**Methods:**

Eleven neonatal rats were generated from heterozygote (ET_B_^+/−^) crossbreeding. Age and bodyweight were recorded at time of sacrifice. Diffusion-staining protocols with 1.5% iodine solution was completed prior to micro-CT scanning. All rats were scanned using an in vivo micro-CT scanner, Caliper Quantum FX, followed by two quality-control scans using a custom-built ex vivo micro-CT system. All scans were reviewed for gross cardiac dysmorphology. Micro-CT data were segmented semi-automatically post-NLM filtering for: whole-heart, LV, RV, LA, RA, and aortic arch. Measurements were taken with Drishti. Following image analysis, PCR genotyping of rats was performed: five *sl/sl* rats, three wildtype, and three heterozygotes. Statistical comparisons on organ volume, growth rate, and organ volume/bodyweight ratios were made between *sl/sl* and the control group.

**Results:**

Cardiac morphology and constituents were preserved. However, significant volumetric reductions were recorded in *sl/sl* rats with respect to the control: whole heart (38.70%, *p value* = 0.02); LV (41.22%, *p value* = 0.01), RV (46.15%, *p value* = 0.02), LA (44.93%, *p value* = 0.06), and RA (39.49%, *p value* = 0.02). Consistent trend was observed in growth rate (~ 20%) and organ-volume/bodyweight ratios (~ 25%). On the contrary, measurements on aortic arch demonstrated no significant difference among the two groups.

**Conclusion:**

Despite the presence of normal morphology, significant cardiac growth retardation was detected in *sl/sl* rat, supporting the likely association of cardiac anomalies with HSCR, at least in ET_B_^−/−^ subtype. Structural reduction was likely due to a combination of failure to thrive from enteric dysfunction, alterations to CaNCC colonization, and importantly coronary hypoperfusion from elevated ET-1/ET_A_-mediated hypervasoconstriction. Little correlation was detected between aortic arch development and *sl/sl* rat, supporting minor ET_B_ role in large vessels. Although further clinical study is warranted, HSCR patients may likely require cardiac assessment in view of potential congenital cardiac defects.

**Supplementary Information:**

The online version contains supplementary material available at 10.1186/s12872-021-02281-2.

## Highlights

- ET_B_^−/−^ HSCR model was associated with body-growth impairment; neonatal *sl/sl* rat has 16% less bodyweight than the control group.
- *sl/sl* rat has a normal cardiac morphology.
- Significant reductions were detected in cardiac structures of *sl/sl* rats: 40% in whole-heart volume, 20% in growth rate, and 25% in whole heart/bodyweight ratios. Similar trend was observed in the measurements of LA, LV, RA, and RV
- No consistent correlation was observed between ET_B_ genotype and aortic arch sizes.
- These findings supported HSCR patients may have various degrees of neonatal cardiac anomalies; wholistic post-operative care should be considered, including screening for cardiac risk factors.
- In consideration of the result from this paper, genotyping study of patients with and without heart failure would be an interesting step study to evaluate possible correlation between ET_B_ genotype and risks of hypertension, ischemic heart disease, exercise capacity, and heart failure.

**Supplementary Information:**

The online version contains supplementary material available at 10.1186/s12872-021-02281-2.

## Background

Endothelin B receptor (ET_B_) is a G-protein-coupled heptahelical receptor sharing the same class as endothelin A receptor (ET_A_). Both receptors are widely expressed with interconnected functions, particularly in the cardiovascular system. Both ET_A_ and ET_B_ initiate downstream signalling through binding to endothelin. Endothelin are 21 amino-acids peptides categorized into three subclasses: ET-1, ET-2, and ET-3 [1]. ET-3 is responsible for the proliferation of pluripotent neural crest cells (NCCs) through its interaction with ET_B_ to ensure normal intestinal development whereas ET-1 and ET-2 exert vascular functions through ET_A_ and ET_B_ binding.

Homozygous ET_B_ mutation is known to cause Hirschsprung’s disease (HSCR), a well-described aganglionic intestinal disease affecting 1/5000 births globally but with a incidence up to 1/1370 births regionally [2]. HSCR is generally treated with surgical resections; however, increasing understanding on HSCR and ET_B_ regulatory signalling suggests such treatments may not be sufficiently comprehensive. Recent studies suggested that multiple organ systems maybe be affected in syndromic HSCR patients [3, 4]; up to 30% of HSCR patients are associated with abnormalities in the central nervous, gastrointestinal, genitourinary, endocrinological, immunological, and cardiovascular systems. Some of the HSCR-associated syndromes with cardiac abnormalities including Down’s syndrome (up to 2–10% of HSCR cases), Di George syndrome, Haddad syndrome, Mowat–Wilson syndrome, Type IV Waardenburg syndrome (WS-IV), and McKusick-Kauffman syndrome (MKKS) [3, 5–7]. Furthermore, conotruncal heart malformations such as atrioseptal defect (ASD; 2.2%) and ventricular septal defect (VSD; 1.7%) are also noted in subpopulations of HSCR patients [8].

The pathogenesis of heart defects associated with HSCR and ET_B_ mutation may partially stem from the migration failure of cardiac neural crest cell (CaNCC), a process dictated predominantly by ET-1/ET_A_ signalling based on current literature [9]. Animals with ET_A_ mutation have been known to have high neonatal mortality with severe craniofacial and cardiac malformations [10, 11]. Although ET_B_ mutation is not known to affect CaNCC directly, it has been reported to alter ET-1/ET_A_ signalling [12]. Consequently, one can expect subtle cardiac changes to occur in ET_B_^−/−^ mutants.

Vascular dysfunction may also predispose ET_B_^−/−^ mutant to suffer to structural changes during development. ET_B_’s cardiovascular effects are bimodal, mediating both vasodilation and vasoconstriction through its binding with ET-1 [13–15], the principal endothelin isoform in the cardiovascular system. ET-1 is secreted by the vascular endothelial cells and endocardial cells of cardiomyocytes [16]. Several studies have demonstrated that activation of ET-1/ET_B_ pathway yields endothelial vasodilation via nitric oxide (NO), prostacyclin, and endothelium-relaxing factor (EDRF) thus balancing the vasoconstriction mediated by ET-1/ET_A_ in vascular smooth muscle cells (VSMC). Consequently, ET_B_ is likely to possess a beneficial role in myocardial circulation and thus end organ perfusion [17–19]. By the same token, one would expect HSCR patients with homozygous ET_B_^−/−^ mutation to have impaired cardiovascular development and increased risks for hypertension and coronary artery disease due to the uncontrolled ET-1/ET_A_ stimulation [20].

Although several microscopic and physiologic studies have been conducted to determine the functions and distribution of ET_B_ receptor, to the best of our knowledge, no macroscopic analyses have been completed to evaluate its effect on cardiac anatomy [4, 15, 21]. We aim to complement prior studies through quantitative analysis on the cardiac changes of the spotting-lethal (*sl/sl*) rat, a naturally occurring ET_B_^−/−^ animal model of WS-IV, with the phenotype of HSCR, hearing deficits, and white coat colour [22].

In order to acquire detailed yet structurally preserved anatomical information, we adopted X-ray micro-computed tomography (micro-CT) with modified tissue staining techniques [23]. Micro-CT offers three-dimensional (3D) information with high resolution images comparable to the low powered 2D microscopy. In addition, improvement on imaging analysis software in recent years have rendered detection of subtle volumetric and dimensional changes in cardiac system possible.

In this study, we hypothesize the following:

1. ET_B_^−/−^ mutant may suffer minor body growth impairment in early age.
2. Gross cardiac morphology may be preserved in ET_B_^−/−^ mutant.
3. However, loss of functional ET_B_ gene may be associated with reduced heart size, growth rate, and heart volume/bodyweight ratio.
4. Cardiac growth may be ET_B_ dose dependent.
5. Absence of functional ET_B_ has little effect on aortic arch growth.

## Methods

### Compliance with ethical practice

All tissues and animals used in this study were handled with care and strict compliance to ACT Health Human Research Ethics Committee (ACTH-HREC) and Australian National University Animal Experimentation Ethics Committee (ANU-AEEC), ethic approval project number A2011/67.

### Rat culling and method of euthanasia

All rat specimens used in this study were generated from crossbreeding between the heterozygous (ET_B_^+/−^) parents. This breeding colony was originally derived from a naturally occurring mutation and has been maintained in Australian National University (ANU) over the past 15 years.

Eleven neonatal rats with an average age of 88 h were sacrificed. Individual rat’s coat pattern, age, gender, and weight were recorded. These rats were over anaesthetized with 5% isoflurane for 15 min in modified gas chamber prior culling. These rats were culled via abdominal aortotomy following a midline laparotomy of 1 cm using scalpel and iris scissor. Five-millimetre tail-tip of each rat was resected and stored for subsequent genotyping.

### Tissue preparation and staining protocols

For successful micro-CT scanning, diffusion staining was performed through the following steps. Firstly, midline thoracotomy of one centimetre was performed on rat carcass to facilitate tissue penetration of staining solution into the cardiac tissue. The thoracotomized bodies were immersed in 10% PBS solution for 30 min to wash out residual bodily fluid. This was followed by tissue fixation in 4% formalin solution for 24 h. Next, formalin-fixed tissues were then washed out with graded ethanol (EtOH) series: 20%, 50%, 70%, and 90% for 1 day each. Finally, tissue staining was completed in 1.5% iodine in 90% EtOH for a minimum of seven days prior to micro-CT scanning.

### Image acquisition by micro-CT scanning

In this study, all rat samples have been scanned using a commercial in vivo micro-CT scanner, Caliper Quantum FX. Additional quality assurance micro-CT scans were acquired using a custom-built ex vivo micro-CT system by ANU Applied Mathematical Department. The maximal resolution achievable by Caliper Quantum FX was 10 µm/voxel with an efficiency of 4.5 min per scan. The average dataset size was 256 MB. The resultant images were stored as DICOM series and visualized using Drishti, an open-source software [24]. The ex vivo micro-CT system built by ANU Applied Mathematical Department required at least fifteen hours of scanning time with additional eight hours of image-processing time via National Computational Infrastructure (NCI) services. The maximal resolution was 1 µm/voxel; magnification factor was limited by the physical size of the sample. The resultant images were stored as netCDF files and visualized with Drishti [24]. Each dataset has an average size of 12–16 GB.

Due to the limited access to the ex vivo micro-CT scanners, all image data acquired by Caliper Quantum FX were filtered with non-local means (NLM) algorithms to improve image quality [25]. Two sets of ex vivo micro-CT data were acquired for quality control to ensure sufficient anatomical details and image clarity in denoised in vivo micro-CT scans available for quantitative analysis. Although not ideal*,* we found image quality of post-processed in vivo micro-CT scans satisfactory for the purposes of this study.

### Image segmentation and analysis

Acquired micro-CT data were first denoised using NLM algorithm to improve signal-to-noise ratios and therefore image clarity [25]. This code was implemented on an Intel (R) Core ™ i7-4770 K CPU @3.5 GHz system with 32G of RAM and Nvidia GeForce GTX Titan Black Kepler GK110 architecture running Linux.

Following image filtering, micro-CT data were segmented semi-automatically through individual CT slices for selected organs using Drishti segmentation algorithms [24]. To ensure accuracy, follow-up verification on image segmentation was performed one month after the initial processing: minimal variations were detected. The following cardiac organs were selected for quantitative measurements: whole heart, left atrium (LA), left ventricle (LV), right atrium (RA), right ventricle (RV), and ascending aortic arch (AA). This process was repeated for each structure. Segmentation of the whole heart was first completed to determine potential gross cardiac defects in with ET_B_^−/−^ mutants. The luminal width of AA was measured at the aortic orifice in axial view for standardize comparison. Three-dimensional (3D) volumetric measurements were completed following sub-segmentation of each structure through volume rendering.

To standardize comparison, the following anatomical definitions were adopted. The pulmonary circulatory inflow was defined by the superior and inferior vena cava orifices to right atrium; the outflow was defined by the pulmonary valve. The systemic circulatory inflow was defined by pulmonary vein orifices to the left atrium whereas the outflow was defined by the aortic valve. Both selections of left and right ventricles have included interventricular septal wall for better definition of organ boundary to standardize comparison. Lastly, for the comparison of AA, the boundary of AA was defined as arterial vessel between the aortic valves to the first branching point, brachiocephalic artery.

### H&E light microscopy

H&E light microscopy was completed for two of eleven rats following micro-CT scanning to assess cardiac anatomy presented in micro-CT scans. The following steps were performed. The iodine-stained hearts were sectioned longitudinally into tissue blocks of 4 mm in thickness and placed in cassettes. Contrast washout and dehydration were performed in 90% EtOH for 48 h prior to paraffin embedment at 60 °C. These tissue blocks were then sliced into 4 µm thick tissue-sheets with a microtome. Tissue-sheets were then laid in water-bath of 5–6 °C while being positioned onto labelled-glass slides. These slides were dried overnight at 37 °C.

Progressive H&E staining was completed by placing the slides in alum-hematoxylin solutions until the appearance of dark red colour. Washing and ‘bluing’ with lithium carbonate solution were then performed. Lastly, washing and counter-staining with 0.5% eosin alcoholic solution were completed.

All H&E slides were reviewed with an Olympus IX71 microscope at 4× magnification.

### Genotyping

After the completion of quantitative measurements, genotyping was completed as described in the following section. Three homozygous wild-type (ET_B_^+*/*+^), three heterozygous (ET_B_^+*/−*^), and five homozygous spotting lethal (*sl/sl*; ET_B_^*−/−*^) rats were identified. The average ages of wild-type, heterozygous, and homozygous mutant rats were 90.7 h, 96 h, and 83.2 h respectively.

PCR genotyping was completed through the following protocols. Five-millimetres tail tips of the eleven rats were lysed using Proteinase K in lysis buffer consisting of 100 mM Tris pH 8, 5 mM EDTA, 0.2% SDS, and 200 mM NaCl in distilled water. The DNA was extracted via vortex heating and centrifuging to separate the DNA-containing supernatants from undigested materials. The supernatants were further vortexed and centrifuged to isolate the DNA pellets. The DNA pellets were then washed with 70% EtOH and dried. The DNA was then suspended and quantified using spectrophotometry. Next, PCR was completed with “Master-Mix” reagent, which included: 10*PCR buffer Qiagen-contained MgCl_2_, dNTP (10 mM), Primer PS7 (33.3 μM; 5′-CCA CTA AGA CCT CCT GGA CT-3′), Primer PS 15 (33.3 μM; 5′-TCA CGA CTT AGA AAG CTA CAC T-3′) and DNA polymerase [26]. Afterward, this Master-Mix reagent was pipetted onto PCR plates filled with the eleven rat DNAs followed by PCR in Veriti 96-Well Thermal-Cycler. Finally, electrophoresis of fourteen DNA samples (eleven test-subject DNA and three controls: wild-type, heterozygote, and *sl/sl* rat) was run for one hour under the voltage setting of 100 V and current setting of 55 mA, with MassRuler (#SM1263, Fermentas) reference by the side. The resultant electrophoresis gel was visualized with Gel Documentation System DOC-Print VX5 (Vilber Lourmat).

### Statistical analysis

Based on the segregation analysis of rat colony data showing strong autosomal recessive inheritance (*p value* = 0.001) and high genetic penetrance (> 95% exhibits HSCR) of *sl/sl* rats, statistical comparison using the two-tail t-test was made between the *sl/sl* (ET_B_^−/−^) and the control group (ET_B_^+/+^ and ET_B_^+/−^) to determine the effect of ET_B_ on heart growth. The comparison was made in the following parameters: organ size, organ growth rate, and organ volume/bodyweight ratios. Albeit small, the latter two were made to further standardize comparisons by accounting age and body size variations of individual rat. Additionally, these parameters provided estimating metrics for structural changes upon developmental maturation.

The difference (%) between the control and *sl/sl* groups were calculated for each parameter. Additionally, the proportionality of individual cardiac substituent with respect to the whole heart (organ/heart ratio) were compared to explore potential regional-dependent effect. Lastly, data of respective wild-types (ET_B_^+/+^) and heterozygotes (ET_B_^+/−^) were provided in the supplementary figures to illustrate of potential gene-dose-dependent relationship.

All statistical analysis was completed one month after the secondary verification review of image segmentation: GraphPad Prism version 8.0.0 for Windows, GraphPad Software, San Diego, California USA, www.graphpad.com.

## Results

### Reduced bodyweight and body growth rate in *sl/sl* rat

**Fig. 1:**
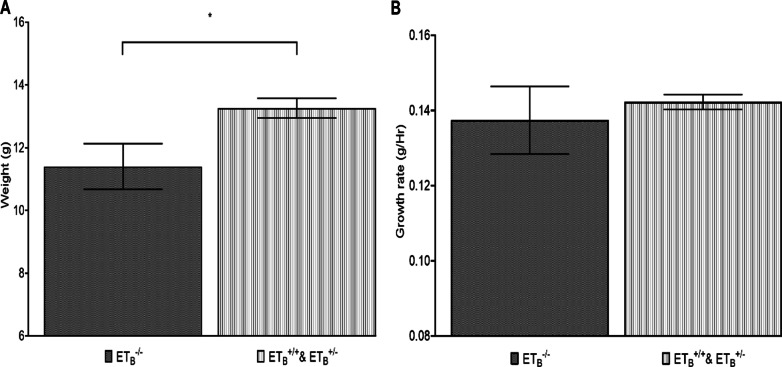
Decreases in bodyweight (**A**) and body-growth rates (**B**) in *sl/sl* rat. **A** showed *sl/sl* (ET_B_^−/−^) rats having statistically significant smaller bodyweight than the control group (ET_B_^+/+^ and ET_B_^+/−^), 11.40 g versus 13.26 g, *p value* = 0.03. However, when corrected with respective age (Hr), smaller difference was observed between the two groups, 0.137 g/h versus 0.142 g/h, *p value* = 0.58. ET_B_^−/−^ induced global growth impairment was likely small in the early stage of development. *: statistically significant, p value ≤ *0.05*

Based on our measurements of neonatal rats, ET_B_ mutation was associated with minor changes in body size and body growth rate, at least at the age of eighty hours. As shown by Fig. 1a, *sl/sl* (ET_B_^−/−^) rats have 16.32% smaller bodyweight of than that of control group, *p value* = 0.03. On the other hand, when standardized to rats’ age, this difference was reduced to 3.53%, *p value* = 0.577, as shown by Fig. 1b. Although not shown, there was little difference in bodyweight and growth rate of the wild-type (ET_B_^+/+^) and heterozygotes (ET_B_^+/−^), suggesting the absence of ET_B_ dose-dependent relationship.

### *sl/sl* rat has grossly normal cardiac morphology

**Fig. 2:**
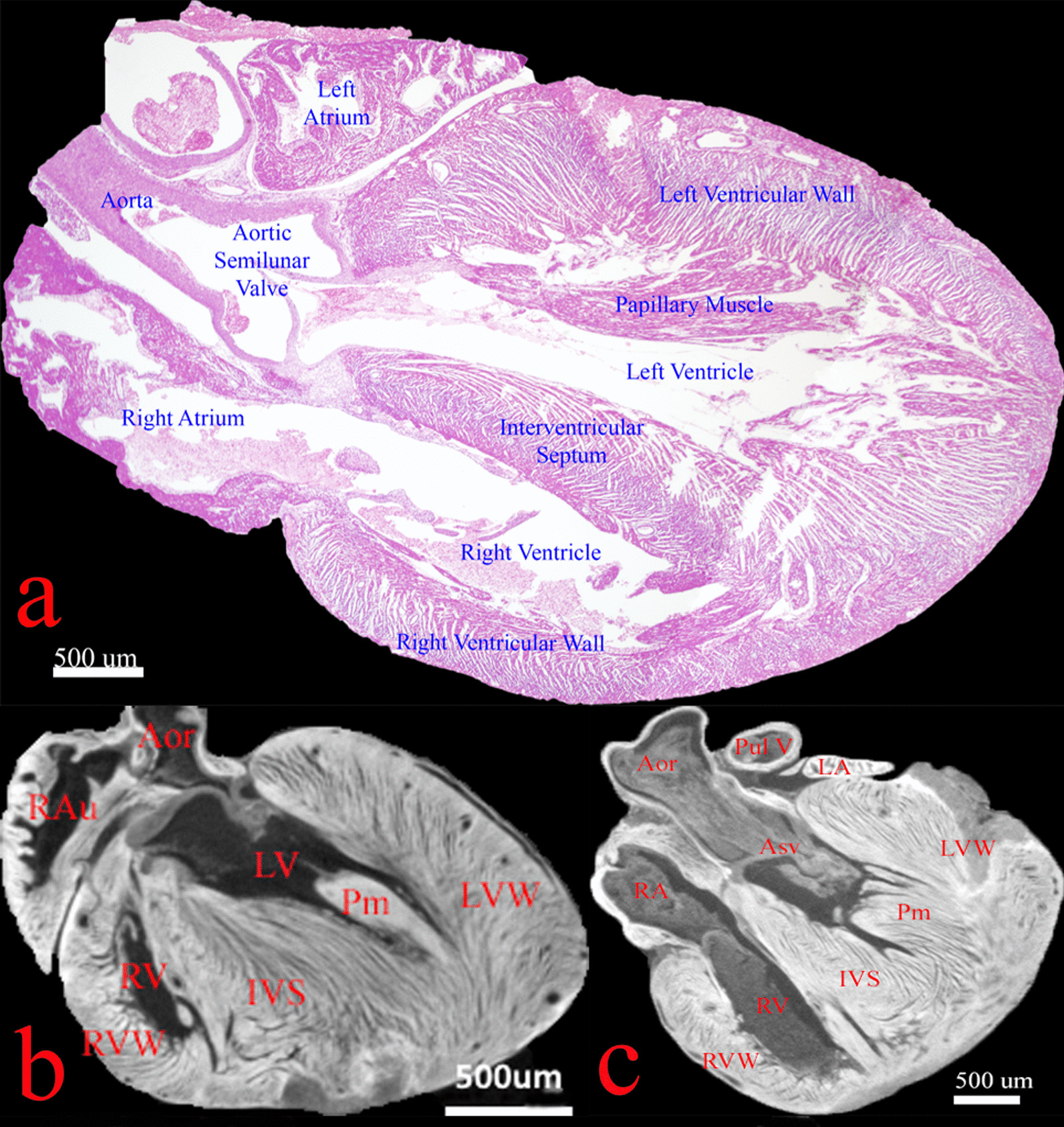
Grossly normal cardiac morphology and constituents in *sl/sl* rat. **A** (4× H&E microscopy), **B** and **C** (ex vivo micro-CT, 10.7 µm/voxel) were cardiac illustrations *sl/sl* rats in coronal views. No detectable gross abnormality was identified. Both image types showed patent cardiac outflow tract with fully formed atria and ventricles. Septal defects were not detected. Major structures were as labelled. *Aor* = *Aorta; RAu* = *Right Auricle; RA* = *Right Atrium; RV* = *Right Ventricle; RVW* = *Right Ventricular Wall; IVS* = *Interventricular Septum; LA* = *Left Atrium; LV* = *Left Ventricle; LVW* = *Left Ventricular Wall; Pm* = *Papillary Muscle; Pul V* = *Pulmonary Vessel; Asv* = *Aortic semilunar valve*

Previous studies have suggested disruptions in the endothelin system may lead to CaNCC migration failure and cause cardiac outflow tract defects [27]. However, thorough reviews of the eleven rat micro-CT scans did not reveal marked dysmorphology in cardiac constituents. As exemplified by the sectional micro-CT slices and H&E scan shown in Fig. 2, *sl/sl* heart contained all the essential components of a normal heart: aorta, aortic semilunar valve, right and left atrium, right and left ventricles, intact interatrial and interventricular septum, and patent pulmonary vessels. Expectedly, cardiac anatomy of wild-type and heterozygous rats shared the same features. This preliminary finding suggested ET_B_ may have little impact on CaNCC migration.

### *sl/sl* rat has significantly smaller heart (mm^3^)

Following the morphological examinations, volumetric measurements were performed for quantitative comparison. Our data demonstrated homozygous ET_B_ mutation was associated with significant volumetric reductions in the heart and constituents.

**Fig. 3:**
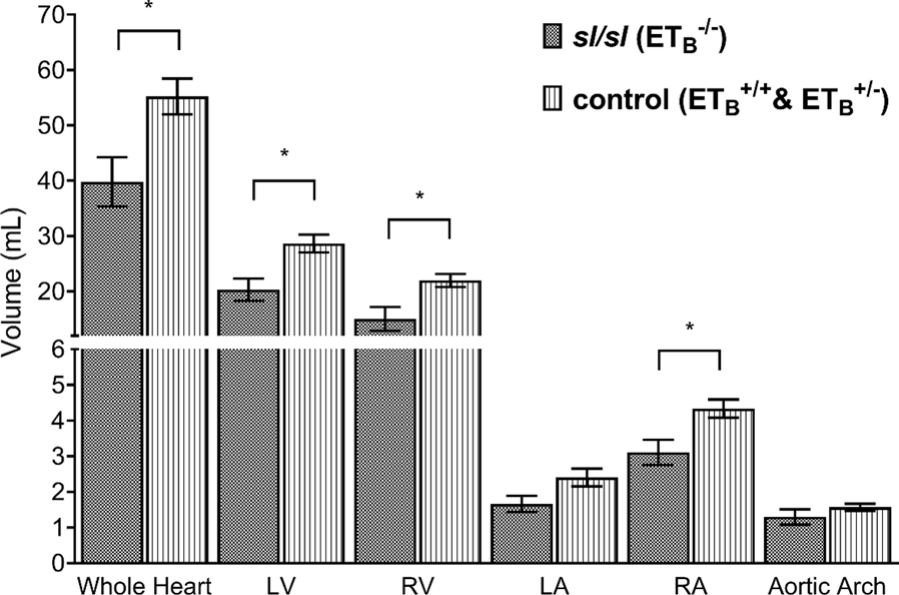
*sl/sl* rat has significantly smaller cardiac structures. *sl/sl* rat has a smaller heart when comparing to the control animals, 39.81mm^3^ versus 55.22mm^3^, *p value* = 0.02. Analyses on heart constituents showed similar trend: LV (20.30mm^3^ vs. 28.67mm^3^, *p value* = 0.01), RV (15.04mm^3^ vs. 21.98mm^3^, *p value* = 0.02), LA (1.66mm^3^ vs. 2.40mm^3^, *p value* = 0.06), and RA (3.11mm^3^ vs. 4.33mm^3^, *p value* = 0.02). Minor volumetric difference in aortic arch was also detected between *sl/sl* and the control group: 1.29 mm^3^ versus 1.56mm^3^, *p value* = 0.25. *RA* = *Right Atrium; RV* = *Right Ventricle; LA* = *Left Atrium; LV* = Left Ventricle. *: statistically significant, p value ≤ *0.05*

As demonstrated by Fig. 3, *sl/sl* rat has a significantly smaller heart than the control group, up to 38.7% reduction, *p value* = 0.02. Similar changes were observed in the following constituents: LV (41.22%, *p value* = 0.01), RV (46.15%, *p value* = 0.02), LA (44.93%, *p value* = 0.06), and RA (39.49%, *p value* = 0.02). Volumetric measurement of AA also showed 22.00% reduction in *sl/sl* rats with respect to the control; however, this finding did not reach statistical significance, *p value* = 0.25.

Although not reported by current literature, cardiac size may be dose-dependent to ET_B_ gene copies. As shown by Additional file 1: Supplementary Fig. 1, wild-type rat has the largest heart among the three genotypes; this was followed by heterozygote in the middle and *sl/sl* rat in the last. Concordantly, LA, RA, LV and RV measurements of wild-types were 42.76–48.09% larger than those of *sl/sl* rats, whereas those of heterozygotes were only 34.71–44.71% larger.

Overall, we demonstrated neonatal *sl/sl* rats having approximately 40% smaller hearts with respect to the control group. These differences may widen with age until rats reach maturity.

### *sl/sl* rat has significantly lower cardiac growth rate (mm^3^/h)

**Fig. 4:**
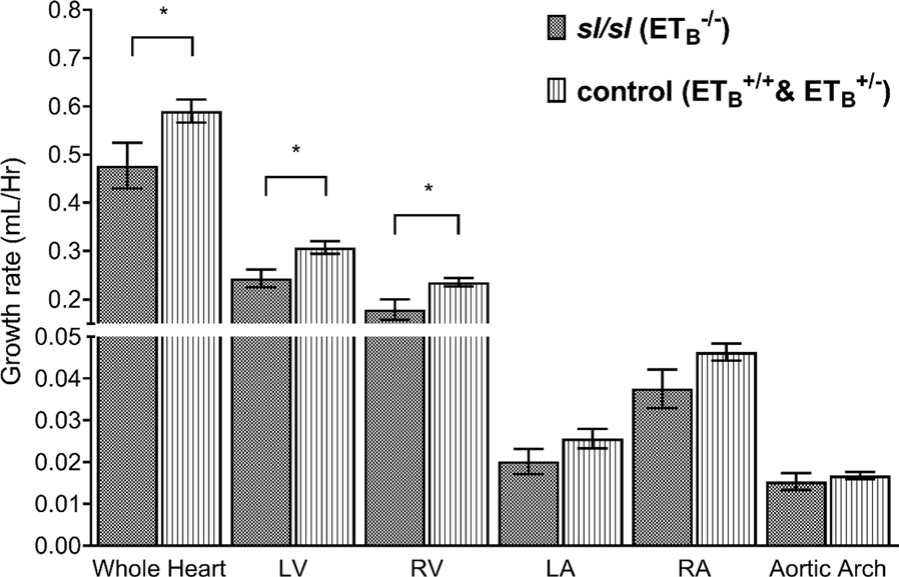
*sl/sl* rat has lower cardiac growth rates than the control group. Markedly lower cardiac growth rate was detected in *sl/sl* rat with respect to the control animal, 0.48 mm^3^/h versus 0.59 mm^3^/h, *p value* = 0.05. Similar difference was observed between the two groups in LV (0.24mm^3^/h vs. 0.31mm^3^/h, *p value* = 0.02) and RV (0.18mm^3^/h vs. 0.23mm^3^/h, *p value* = 0.03). Albeit not reaching statistical power, decreasing trend was also seen in LA (0.020mm^3^/h vs. 0.026mm^3^/h, *p value* = 0.18) and RA (0.038mm^3^/h vs. 0.046mm^3^/h, *p value* = 0.10). Aortic arch measurement showed little difference between the two groups: 0.015mm^3^/h versus 0.017mm^3^/h, *p value* = 0.51. *RA* = *Right Atrium; RV* = *Right Ventricle; LA* = *Left Atrium; LV* = Left Ventricle. *: statistically significant, p value ≤ *0.05*

ET_B_ mutation has a negative impact on cardiac growth rate. As shown by Fig. 4, the growth rate of whole heart in *sl/sl* rat was 23.70% lower than that of control group, *p value* = 0.05. Significant reductions were also observed in the growth rates of LV (26.39%, *p value* = 0.02) and RV (31.25%, *p value* = 0.03). Although not reaching the statistical power, lower growth rates of LA (27.36%, *p value* = 0.18), RA (23.42%, *p-value* = 0.10) and AA (9.28%, *p value* = 0.51) were also detected in the *sl/sl* rats.

Further analysis showed stepwise decreases in organ growth rates with respect to functional copies of ET_B_ gene. As shown by Additional file 2: Supplementary Fig. 2, LA, RA, LV, and RV growth rates of heterozygous rats were 17.40–26.60% higher than those of *sl/sl* rats, whereas those of wild-types were 29.43–35.91% higher. On the other hand, analyses on AA growth rates demonstrated no consistent correlations with ET_B_ dose.

Overall, an approximately 25% decrease in the growth rates of all heart constituents could be appreciated in neonatal *sl/sl* rats. This growth retardation may persist until age of maturation. Interestingly, this reduction was disproportionally larger than the 3.53% reduction in bodyweight growth rate, suggesting an intrinsic effect of ET_B_ to the developing heart.

### *sl/sl* rat has lower heart organ volume/bodyweight indices

**Fig. 5:**
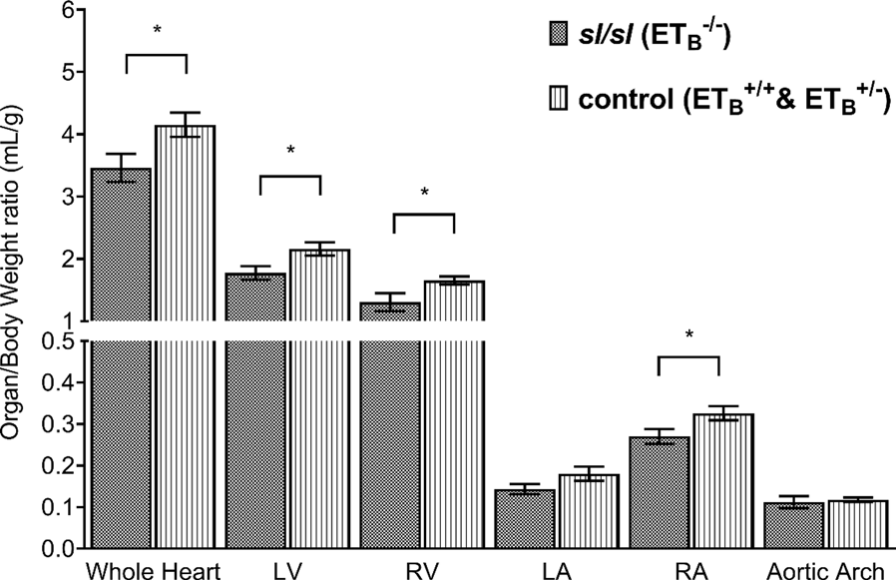
*sl/sl* rat has disproportionally larger reductions in its heart size with respect to its bodyweight. Markedly smaller cardiac-organ/bodyweight ratios were observed in *sl/sl* rat when comparing to those of control group: whole heart (3.46 mL/g vs. 4.15 mL/g, *p value* = 0.04), LV (1.77 mL/g vs. 2.16 mL/g, *p value* = 0.03), RV (1.31 mL/g vs. 1.65 mL/g, *p value* = 0.04), LA (0.14 mL/g vs. 0.18 mL/g, *p value* = 0.13), and RA (0.27 mL/g vs. 0.33 mL/g, *p value* = 0.049). This trend reflected disproportionally larger structural impairment in *sl/sl* heart with respect to global growth restriction, supporting an intrinsic effect of ET_B_ on heart growth. On the contrary, measurement on aortic arch showed little difference between the two groups: 0.112 mL/g versus 0.118 mL/g, *p value* = 0.70. *RA* = *Right Atrium; RV* = *Right Ventricle; LA* = *Left Atrium; LV* = Left Ventricle. *: statistically significant in comparison to ET_*B*_^*−/−*^* group, p* ≤ *0.05*

Cardiac organ volume/bodyweight ratios demonstrated that effect of ET_B_ mutation on heart structure was disproportionally larger than its effect on body size. Figure 5 showed *sl/sl* rat has approximately 20% smaller organ volume/bodyweight ratios than those of the control group in the following: whole heart (20.00%, *p value* = 0.04), RA (20.79%, *p value* = 0.05), LV (21.75%, *p value* = 0.03), and RV (26.54%, *p value* = 0.04). Although not achieving statistical significance, LA volume/bodyweight ratio of *sl/sl* rat also has a reduction of 25.75%, *p value* = 0.13. Overall, homozygous ET_B_ mutation was associated with a disproportionally larger reduction in cardiac sizes with respect to changes in global body size. This was further supported by a stepwise decreasing pattern with reducing copies of functional ET_B_ gene, as illustrated by Additional file 3: Supplementary Fig. 3. On the other hand, little change was detected in AA measurements, with *sl/sl* rat having 5.17% larger AA than that of the control group, *p value* = 0.70; this observation was consistent with ET_B_’s minor regulatory role on large vessel.

### Uniform structural changes associated with ET_B_ mutation

**Fig. 6:**
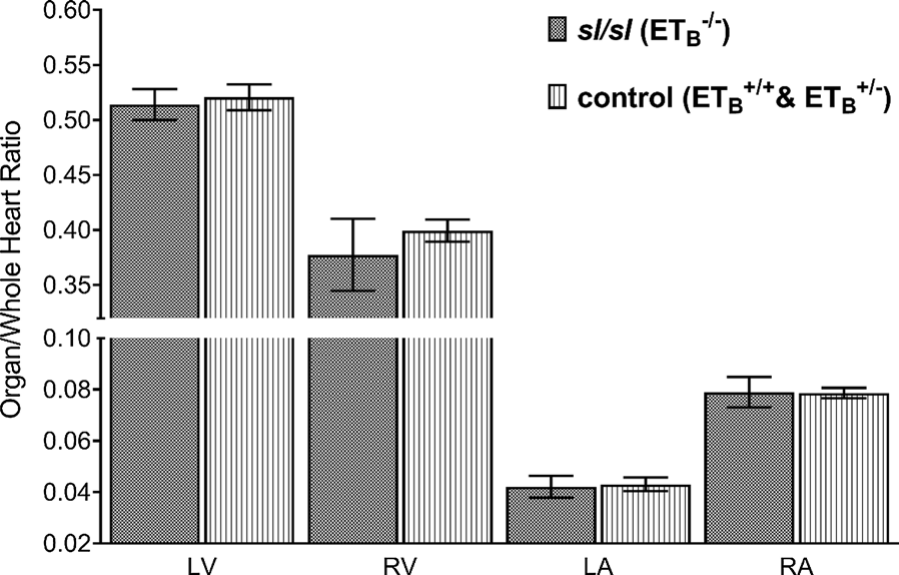
Cardiac shrinkage in *sl/sl* rat was relatively uniform across four major constituents. No significant difference was detected in cardiac constituent/whole heart ratios between *sl/sl* and the control group. Both shared similar organ/whole heart ratios: LV (0.51 vs. 0.52, *p value* = 0.72), RV (0.38 vs. 0.40, *p value* = 0.50), LA (0.042 vs. 0.043, *p value* = 0.95), and RA (0.079 vs. 0.079, *p value* = 0.95). This reflects ET_B_ effect was overall uniform across all heart structures, albeit minor regional-dependent impact might be present in RV

Subtle variations in the structural changes of different heart components were detected in *sl/sl* rat. The differences in cardiac constituent/whole heart ratios were relatively uniform between *sl/sl* rat and the control group, as showed by Fig. 6, suggesting the effect of ET_B_ mutation on the heart was likely global. Overall, these differences between the two groups were insignificant albeit a 5.81% reduction was detected in the RV/whole heart ratio of *sl/sl* rat.

## Discussion

In this study, we demonstrated neonatal ET_B_^−/−^ HSCR model exhibits significant cardiac structural impairment. This was consistently illustrated through three parameters taken in *sl/sl* rat: up to 40% volumetric reductions in heart structures, 20% reduction in heart growth rate, and 25% reduction in organ volume/bodyweight indices. The causes for these cardiac growth restrictions likely included the following: global growth impairments due to enteric dysfunction; alterations in CaNCC development; vasodysregulation caused by the absence of ET_B_.

Concurrent developmental anomalies occurred in up to 30% of HSCR patients, five to eight percent of whom suffer congenital heart diseases (CHD) [28]. While CHD occurred in only three percent of non-syndromic HSCR infants, the prevalence of CHD in chromosomal HSCR patients is remarkably high, ranging from 20 to 80%, with cardiac septal defects being the most common anomalies [5]. Furthermore, regional paediatric data demonstrated that up to 48% of HSCR patients co-affected with Down’s syndrome (HSCR/DS) also have CHD [29]; this high congruence suggested defects in cardiac and central nervous systems may be closely related in certain HSCR subtypes. Indeed, in addition to the cardiac structural abnormalities reported in this study, we have previously found brain shrinkage (up to 20% reduction) in *sl/sl* rat despite preservation of normal organ morphology. Our findings suggested HSCR patients, at least in ET_B_^−/−^ variant, are likely to share subtle cardiac and neurological impairments. This also suggested that CHD incidence in HSCR is likely underestimated due to under-reporting of subtle cardiac anomalies.

Homozygous ET_B_ knock-out mutation (ET_B_^−/−^) causes WS-IV syndrome with prominent HSCR phenotypes. As previously mentioned, aberrant mutation in ET-3/ET_B_ signalling causes ENCC migration failure and thus ENS maldevelopment, which impairs the nutritional absorption in affected individuals [30]. Consistently, a 16.53% decrease in bodyweight and a 3.42% decrease in body growth-rate were recorded in *sl/sl* rat, Fig. 1. These bodily reductions have likely contributed, at least partially, to the proportional shrinkage of the heart. Additionally, this growth retardation will likely worsen with age as the manifestation of enteric failure becomes progressively prominent.

Disruption in CaNCC migration may have contributed to the structural changes of the heart in *sl/sl* rat. Although limited literature is available to comment on the presence of direct linkage between ET-3/ET_B_ signalling on CaNCC migration, indirect alteration in ET-1/ET_A_ signalling through elevated ET-1 levels in ET_B_^−/−^ mutants has been suggested [12]. CaNCC has been documented to colonize cardiac outflow tract (OFT) and pharyngeal arches during embryogenesis. This in turn facilitates the remodelling of pharyngeal arch arteries (PAAs), which gives rise to bilateral carotid arteries, segment of aortic arches, pulmonary artery, and ductus arteriosus [10]. Consequently, removal of CaNCC during development causes inappropriate PAA regression and leads to type b interrupted aortic arch in mouse models [10]. Furthermore, prior studies demonstrated the presence of CaNCC promotes cardiac septation in mouse [31] while its absence leads to ventricular septal defects (VSDs) [11]. Importantly, Clouthier et al. [9] demonstrated mice with defective ET-1/ET_A_ signalling share features of velocardiofacial syndrome like those of CaNCC migration failure. On the other hand, other than significant size shrinkage, *sl/sl* rat seemed to exhibit a grossly normal heart; neither large vessel nor cardiac septal anomaly was detected. Although prior lineage-tracing studies did not reveal the participation of CaNCC in the developments of myocardium and epicardium [32], biomarker studies using *Plxna2*, fate-mapping, and the finding of thin ventricular myocardium as a result of CaNCC gene knock-out studies (e.g. BMPR1A and PAX3) suggested CaNCC contributes to epicardial developments and ventricular myocardium [33–35]. Concordantly, we found marked atrial and ventricular shrinkage in the heart of *sl/sl* rat, supporting subtle alterations in CaNCC pathway from ET-1/ET_A_ hyperstimulation may have partially contributed to the myocardium maldevelopment.

Disruption in ET_B_’s vascular control may have also contributed to the detrimental effect of cardiac development in *sl/sl* rat. ET_B_ is predominantly expressed in the vascular endothelium where it initiates vasodilation through binding with ET-1, thus triggering decreased clearance of NO, prostacyclin, and EDRF. On the other hand, subtle ET_B_ presence has also been detected in VSMC, where activation by ET-1 causes vascular constriction; albeit this effect is minor in normal physiological state [14, 17, 18, 36]. Nilsson et al. [37] illustrated ET_B_’s functional duality through stronger recordings of vasoconstrictive response in endothelium-denuded porcine coronary artery following stimulation by Sarafotoxin 6c. This showed endothelial ET_B_ partially regulates baseline vasodilation and basal coronary perfusion, which are vital to the developing heart. Adding insult to injury, the absence of ET_B_ markedly reduces the clearance of ET-1 and thus increases ET-1 level by up to 6-folds in *sl/sl* rats [12]. This leads to elevated ET-1/ET_A_ stimulation and subsequent hyper-vasoconstriction of coronary artery. Consequently, coronary hypoperfusion worsens growth retardation of the heart, as shown by Figs. 3, 4 and 5 [38]. Interestingly, the pattern of stepwise reduction in heart volume, growth rates, organ volume/bodyweight ratios correspond well with decreasing functional ET_B_ copies, as shown by Additional files 1, 2 and 3: Supplementary Fig. 1–3. This pattern was also consistent with the inverse relationship between myocardial perfusion and the concentration of ET-1 perfusate to coronary artery [38]. Furthermore, Fig. 6 illustrated reduction in ventricular myocardium was slightly more prominent than that of atrial myocardium, which may reflect variance in rat coronary arterial distribution; albeit this regional difference was very small.

Lastly, three-dimensional analyses made on aortic arch demonstrated no conclusive ET_B_-dependent relationship, as shown by Figs. 3, 4 and 5. On the other hand, a subtle decrease in aortic luminal diameter was observed in *sl/sl* rat, as noted by Additional file 4: Supplementary Table 1. This reflected the likely elevated basal vasoconstriction at the time of culling, mediated by elevated ET-1/ET_A_ signalling. Additionally, this finding was consistent with prior reports that contractile control in large vessels is predominantly ET_A_-mediated [13].

Overall, our result showed distinctive difference in cardiac growth between *sl/sl* and the control groups. The effect of ET_B_ on cardiogenesis was likely multifactorial rather than a pure manifestation of HSCR’s poor growth [30]. While we cannot be certain on the exact pathogenesis of ET_B_ dysfunction leading to the cardiac reduction, in conjunction with prior studies, hypoperfusion from vascular dysregulation seems likely. Nevertheless, we acknowledged the possibility of growth impairment from enteric dysfunction and changes in CaNCC colonization to the developing heart, albeit these effects were likely minor if presented. Importantly, our finding provided a clue to the suspected cardiac impairment associated with HSCR, a traditionally thought surgical disease. Indeed, if this quantitative finding is translatable to human, HSCR patients are likely to suffer a significant cardiac structural reduction, ranging from 20 to 40%, and thereby increases risk for development of cardiac failure, at least in the ET_B_^−/−^ subtype. Consequently, a wholistic management may be warranted.

Our findings suggested early screening for cardiovascular risk factors in individuals with HSCR may be beneficial, especially in those with ET_B_^−/−^ subtype. In conjunction to the known vascular dysfunction related to ET_B_^−/−^ mutation, significant structural compromise of the heart in animal model suggested a potential risk for cardiac dysfunction. Thus, a structural review using cardiac magnetic resonance imaging (MRI) followed by a baseline echocardiogram may be beneficial. Additionally, clinical uses of endothelin receptor antagonists should be with increasing caution given the potential adverse effect of ET_B_ inhibition.

## Limitations

Although we have demonstrated significant structural defects in ET_B_^−/−^ HSCR animals, further studies are required. Firstly, we acknowledge the statistical power can be improved by higher sampling; this can be achieved once a more efficient image segmentation platform becomes available. Secondly, despite image analysis was completed on a pre-set computing algorithm to minimize potential bias, future study should consider adopting additional operators and various software for verification of findings. Furthermore, functional assessments with echocardiograms may be beneficial in establishing the direct correlations between cardiac dysfunction and structural impairments. Lastly, clinical study using cardiac MRI can be beneficial in confirming cardiac defects in HSCR patients and should be considered in future studies.

## Conclusion

This study demonstrated a correlation between ET_B_ function and cardiac size in the *sl/sl* rat. The exact mechanism of action is unclear at this point but may well be due to an effect on coronary artery tone. Preserved cardiac morphology and aortic arch dimensions suggest impaired CaNCC migration failure may play little role in HSCR. Nevertheless, the finding of significant cardiac size and growth impairment in HSCR model is consistent with an elevated risk of congenital structural heart disease in HSCR patients. It also raises the possibility that ET_B_ gene may have a role in the development of heart failure, coronary artery disease, and hypertension in the general population.

## Supplementary Information

**Additional file 1**: Figure 1. Stepwise structural shrinkages were associated with decreasing functional ET_B_. Stepwise reduction in cardiac size was associated with decreasing ET_B_ copies, with wild-type having largest heart and constituents, followed by heterozygotes in the middle, and *sl/sl* having the smallest structures: whole heart (55.83 mm^3^; 54.61 mm^3^; 39.81 mm^3^), LV(29.68 mm^3^; 27.66 mm^3^; 20.30 mm^3^), RV (22.19 mm^3^; 21.76 mm^3^; 15.04 mm^3^), LA (2.46 mm^3^; 2.35 mm^3^; 1.66 mm^3^), and RA (4.43 mm^3^; 4.23 mm^3^; 3.11 mm^3^). The difference between wild-type and heterozygotes was relatively small whereas tissue shrinkages in *sl/sl* rat were markedly larger. On the other hand, aortic arch measurements did not suggest gene-dose-dependency: wild-type (1.39 mm^3^), heterozygotes (1.74 mm^3^), and sl/sl (1.29 mm^3^). RA = Right Atrium; RV = Right Ventricle; LA = Left Atrium; LV = Left Ventricle. *: statistically significant in comparison to ET_B_^−/−^ *group, p ≤ 0.05*.

**Additional file 2**: Figure 2. Stepwise growth rate reduction was associated with decreasing functional ET_B_. Stepwise decrease in cardiac growth rates corresponded to reducing ET_B_ copies. As shown, wild-type has highest growth rates, followed by heterozygotes in the middle, and lowest in sl/sl rat: whole heart (0.61 mm^3^/Hr; 0.57 mm^3^/Hr; 0.48 mm^3^/Hr), LV(0.33 mm^3^/Hr; 0.29 mm^3^/Hr; 0.24 mm^3^/Hr), RV (0.24 mm^3^/Hr; 0.23 mm^3^/Hr; 0.18 mm^3^/Hr), LA (0.027 mm^3^/Hr; 0.024 mm^3^/Hr; 0.020 mm^3^/Hr), and RA (0.049 mm^3^/Hr; 0.044 mm^3^/Hr; 0.038 mm^3^/Hr). Wild-type and heterozygotes have relatively small growth rate variations when comparing their respective differences to sl/sl rat. No consistent correlations with genotype can be deduced from aortic arch measurements: wild-type (0.015 mm^3^/Hr), heterozygotes (0.018 mm^3^/Hr), and sl/sl (0.015 mm^3^/Hr). RA = Right Atrium; RV = Right Ventricle; LA = Left Atrium; LV = Left Ventricle. *: statistically significant in comparison to ET_B_^−/−^ *group, p ≤ 0.05*.

**Additional file 3**: Figure 3. stepwise reduction in organ/bodyweight ratio associated with decreasing copies of functional ET_B_. Positive correlations between cardiac organ/bodyweight indices and ET_B_ gene copy was demonstrated. Wild-type has the highest ratio, followed by heterozygotes in the middle, and *sl/sl* having the lowest: whole heart (4.31 mm^3^/g; 4.00 mm^3^/g; 3.46 mm^3^/g), LV(2.30 mm^3^/g; 2.02 mm^3^/g; 1.77 mm^3^/g), RV (1.71 mm^3^/g; 1.59 mm^3^/g; 1.31 mm^3^/g), LA (0.19 mm^3^/g; 0.17 mm^3^/g; 0.14 mm^3^/g), and RA (0.34 mm^3^/g; 0.31 mm^3^/g; 0.27 mm^3^/g). Minor difference was presented between wild-type and heterozygote when comparing to the reductions observed in sl/sl rats. In conjunction with Figure 1, this supported ETB mutation has intrinsic effect on cardiac development. No consistent trend associating with genotype can be deduced from aortic arch measurements: wild-type (0.11 mm^3^/g), heterozygotes (0.13 mm^3^/g), and sl/sl (0.11 mm^3^/g). RA = Right Atrium; RV = Right Ventricle; LA = Left Atrium; LV = Left Ventricle. *: statistically significant in comparison to ET_B_^−/−^ *group, p ≤ 0.05*.

**Additional file 4. Supplementary Table 1:** sl/sl rat has reduced luminal width of aortic arch when comparing to the control groups. Two-dimensional measurement at the entry of aortic valve showed sl/sl rat having smaller intravascular width comparing to the control group. This trend persisted when respective age and bodyweight were standardized, as shown by the comparison of width growth-rate and width/bodyweight ratio, suggesting possible vasoconstriction.

## Data Availability

Due to the significantly large micro-CT file size for public repositories, all datasets are stored in the National Computational Infrastructure (NCI) Australia and available upon request. Request may be sent directly to corresponding author at ckochin@gmail.com.

## Abbreviations

AA: Aortic arch
ANU-AEEC: Australian National University Animal Experimentation Ethics Committee
ACTH-HREC: ACT Health Human Research Ethics Committee
ASD: Atrioseptal defect
CaNCC: Cardiac neural crest cell
CAS: Cardiovascular system
CHD: Congenital heart disease
CNCC: Cephalic neural crest cell
CNS: Central nervous system
DICOM: Digital imaging and communications in medicine
DNA: Deoxyribonucleic acid
dNTP: Deoxynucleoside triphosphate
DS: Down’s syndrome
GDNF: Glial cell line derived neurotrophic factor
GFRα1: GDNF family receptor alpha 1
GI: Gastrointestinal
H&E: Hematoxylin and eosin stain
HSCR: Hirschsprung’s disease
ECE-1: Endothelin converting enzyme-1
ECE-2: Endothelin converting enzyme-1
EDRF: Endothelium-relaxing factor
EDTA: Ethylenediaminetetraacetic acid
ENCC: Enteric neural crest cell
ET-1: Endothelin-1
ET-2: Endothelin-2
ET-3: Endothelin-3
ET_A_: Endothelin-A receptor
ET_B_: Endothelin-B receptor
EtOH: Ethanol
LA: Left atrium
LV: Left ventricle
MgCl_2_: Magnesium chloride
Micro-CT: Micro-computed tomography
MKKS: McKusick–Kauffman syndrome
MRI: Magnetic resonance imaging
NaCl: Sodium chloride
NCC: Neural crest cells
NCI: National computational infrastructure
NLM: Non-local means
NO: Nitric oxide
OFT: Outflow tract
PAA: Pharyngeal arch artery
PCR: Polymerase chain reaction
RA: Right atrium
RV: Right ventricle
RET: Proto-oncogene RET
*sl/sl*: Spotting-lethal
3D: Three-dimensional
2D: Two-dimensional
VSD: Ventricular septal defect
VSMC: Vascular smooth muscle cell
WS-IV: Waardenburg–Shah syndrome

## References

[1] Kedzierski RM, Yanagisawa M (2001). Endothelin system: the double-edged sword in health and disease. Annu Rev Pharmacol Toxicol.

[2] Meza-Valencia BE, de Lorimier AJ, Person DA. Hirschsprung disease in the U.S. associated Pacific Islands: more common than expected. Hawaii Med J. 2005;64(4):96–8, 100–1. 15921246

[3] Ida-Eto M, Ohgami N, Iida M, Yajima I, Kumasaka MY, Takaiwa K (2011). Partial requirement of endothelin receptor B in spiral ganglion neurons for postnatal development of hearing. J Biol Chem.

[4] Hua LL, Vedantham V, Barnes RM, Hu J, Robinson AS, Bressan M (2014). Specification of the mouse cardiac conduction system in the absence of Endothelin signaling. Dev Biol.

[5] Duess JW, Puri P (2015). Syndromic Hirschsprung's disease and associated congenital heart disease: a systematic review. Pediatr Surg Int.

[6] Sandoval RL, Zaconeta CM, Margotto PR, Cardoso MT, Franca EM, Medina CT (2016). Congenital central hypoventilation syndrome associated with Hirschsprung's Disease: case report and literature review. Rev Paul Pediatr.

[7] Davenport M, Taitz LS, Dickson JA (1989). The Kaufman-McKusick syndrome: another association. J Pediatr Surg.

[8] Spouge D, Baird PA (1985). Hirschsprung disease in a large birth cohort. Teratology.

[9] Clouthier DE, Hosoda K, Richardson JA, Williams SC, Yanagisawa H, Kuwaki T (1998). Cranial and cardiac neural crest defects in endothelin-A receptor-deficient mice. Development.

[10] Kirby ML, Gale TF, Stewart DE (1983). Neural crest cells contribute to normal aorticopulmonary septation. Science.

[11] Porras D, Brown CB (2008). Temporal–spatial ablation of neural crest in the mouse results in cardiovascular defects. Dev Dyn Off Publ Am Assoc Anat.

[12] Gariepy CE, Ohuchi T, Williams SC, Richardson JA, Yanagisawa M (2000). Salt-sensitive hypertension in endothelin-B receptor-deficient rats. J Clin Investig.

[13] Bacon CR, Cary NR, Davenport AP (1996). Endothelin peptide and receptors in human atherosclerotic coronary artery and aorta. Circ Res.

[14] Seo B, Oemar BS, Siebenmann R, von Segesser L, Luscher TF (1994). Both ETA and ETB receptors mediate contraction to endothelin-1 in human blood vessels. Circulation.

[15] Pollock DM, Portik-Dobos V, Procter C, Gariepy CE, Yanagisawa M (2000). Arterial pressure response to endothelin-1 and sarafotoxin 6c in rescued endothelin-B-deficient rats. J Cardiovasc Pharmacol.

[16] Saetrum Opgaard O, Adner M, Peters TH, Xu CB, Stavenow L, Gulbenkian S (2001). Endocardial expression and functional characterization of endothelin-1. Mol Cell Biochem.

[17] Hirata Y, Emori T, Eguchi S, Kanno K, Imai T, Ohta K (1993). Endothelin receptor subtype B mediates synthesis of nitric oxide by cultured bovine endothelial cells. J Clin Investig.

[18] Namiki A, Hirata Y, Ishikawa M, Moroi M, Aikawa J, Machii K (1992). Endothelin-1- and endothelin-3-induced vasorelaxation via common generation of endothelium-derived nitric oxide. Life Sci.

[19] de Nucci G, Thomas R, D'Orleans-Juste P, Antunes E, Walder C, Warner TD (1988). Pressor effects of circulating endothelin are limited by its removal in the pulmonary circulation and by the release of prostacyclin and endothelium-derived relaxing factor. Proc Natl Acad Sci USA.

[20] Kelland NF, Webb DJ (2007). Clinical trials of endothelin antagonists in heart failure: publication is good for the public health. Heart (British Cardiac Society).

[21] Skovsted GF, Kruse LS, Larsen R, Pedersen AF, Trautner S, Sheykhzade M (2014). Heart ischaemia-reperfusion induces local up-regulation of vasoconstrictor endothelin ETB receptors in rat coronary arteries downstream of occlusion. Br J Pharmacol.

[22] Gariepy CE, Cass DT, Yanagisawa M (1996). Null mutation of endothelin receptor type B gene in spotting lethal rats causes aganglionic megacolon and white coat color. Proc Natl Acad Sci USA.

[23] Chen KCAA, Song ZM, Croaker DG (2018). High-definition heart visualization using micro-CT scanning on experimental rats. J Clin Exp Cardiolog.

[24] Limaye AD. A volume exploration and presentation tool. Developments in X-Ray Tomography VIII; 17 October 20122012.

[25] Buades A, Coll B, Morel J-M. A Non-local algorithm for image denoising. In: Proceedings of the 2005 IEEE computer society conference on computer vision and pattern recognition (CVPR'05)—volume 2—volume 02. 1069066: IEEE Computer Society; 2005. pp. 60–5.

[26] Brennan A, Dean CH, Zhang AL, Cass DT, Mirsky R, Jessen KR (2000). Endothelins control the timing of schwann cell generation in vitro and in vivo. Dev Biol.

[27] Kurihara Y, Kurihara H, Oda H, Maemura K, Nagai R, Ishikawa T (1995). Aortic arch malformations and ventricular septal defect in mice deficient in endothelin-1. J Clin Investig.

[28] Pini Prato A, Rossi V, Mosconi M, Holm C, Lantieri F, Griseri P (2013). A prospective observational study of associated anomalies in Hirschsprung's disease. Orphanet J Rare Dis.

[29] Croaker GDH (2002). Clinical and molecular biological studies in Hirschsprung's disease [Doctoral thesis, University of Sydney, Sydney, Australia].

[30] Dembowski C, Hofmann P, Koch T, Kamrowski-Kruck H, Riedesel H, Krammer HJ (2000). Phenotype, intestinal morphology, and survival of homozygous and heterozygous endothelin B receptor–deficient (spotting lethal) rats. J Pediatr Surg.

[31] Webb S, Qayyum SR, Anderson RH, Lamers WH, Richardson MK (2003). Septation and separation within the outflow tract of the developing heart. J Anat.

[32] Jiang X, Rowitch DH, Soriano P, McMahon AP, Sucov HM (2000). Fate of the mammalian cardiac neural crest. Development.

[33] Brown CB, Feiner L, Lu MM, Li J, Ma X, Webber AL (2001). PlexinA2 and semaphorin signaling during cardiac neural crest development. Development.

[34] Engleka KA, Gitler AD, Zhang M, Zhou DD, High FA, Epstein JA (2005). Insertion of Cre into the Pax3 locus creates a new allele of Splotch and identifies unexpected Pax3 derivatives. Dev Biol.

[35] Stottmann RW, Choi M, Mishina Y, Meyers EN, Klingensmith J (2004). BMP receptor IA is required in mammalian neural crest cells for development of the cardiac outflow tract and ventricular myocardium. Development.

[36] Tirapelli CR, Casolari DA, Yogi A, Montezano AC, Tostes RC, Legros E (2005). Functional characterization and expression of endothelin receptors in rat carotid artery: involvement of nitric oxide, a vasodilator prostanoid and the opening of K+ channels in ETB-induced relaxation. Br J Pharmacol.

[37] Nilsson D, Wackenfors A, Gustafsson L, Ugander M, Paulsson P, Ingemansson R (2008). Endothelin receptor-mediated vasodilatation: effects of organ culture. Eur J Pharmacol.

[38] Domenech R, Macho P, Gonzalez R, Huidobro-Toro JP (1991). Effect of endothelin on total and regional coronary resistance and on myocardial contractility. Eur J Pharmacol.

