# Supplementary figures and images for: Structural heart defects associated with ETB mutation, a cause of Hirschsprung disease
Source: BMC Cardiovasc Disord. 2021 Oct 2;21:475. doi: 10.1186/s12872-021-02281-2 (PMC8487587; doi:10.1186/s12872-021-02281-2)

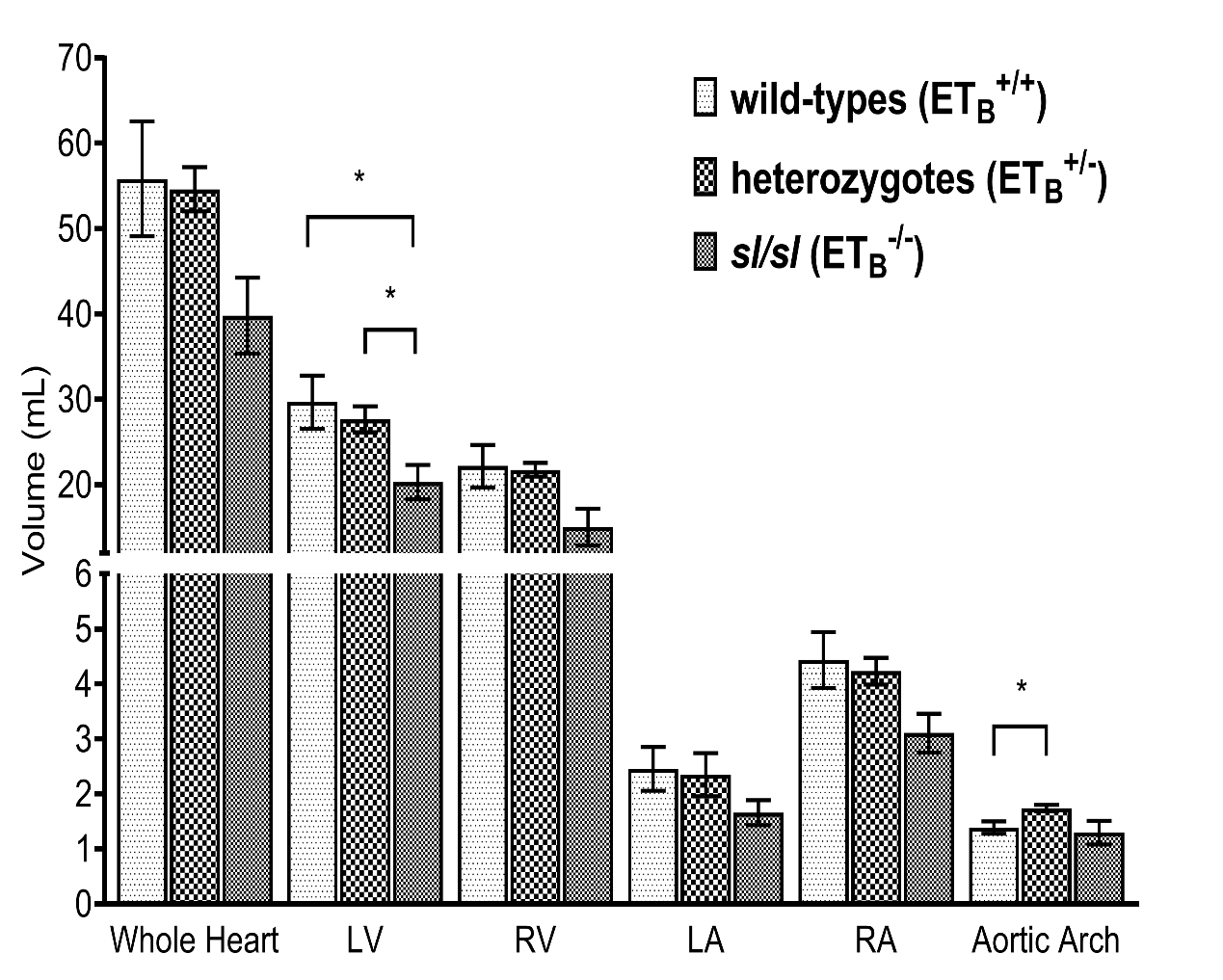

Supplement: Supplementary file 1 — Additional file 1: Figure 1. Stepwise structural shrinkages were associated with decreasing functional ETB. Stepwise reduction in cardiac size was associated with decreasing ETB copies, with wild-type having largest heart and constituents, followed by heterozygotes in the middle, and sl/sl having the smallest structures: whole heart (55.83 mm3; 54.61 mm3; 39.81 mm3), LV(29.68 mm3; 27.66 mm3; 20.30 mm3), RV (22.19 mm3; 21.76 mm3; 15.04 mm3), LA (2.46 mm3; 2.35 mm3; 1.66 mm3), and RA (4.43 mm3; 4.23 mm3; 3.11 mm3). The difference between wild-type and heterozygotes was relatively small whereas tissue shrinkages in sl/sl rat were markedly larger. On the other hand, aortic arch measurements did not suggest gene-dose-dependency: wild-type (1.39 mm3), heterozygotes (1.74 mm3), and sl/sl (1.29 mm3). RA = Right Atrium; RV = Right Ventricle; LA = Left Atrium; LV = Left Ventricle. *: statistically significant in comparison to ETB−/− group, p ≤ 0.05. [file 12872_2021_2281_MOESM1_ESM.tif]

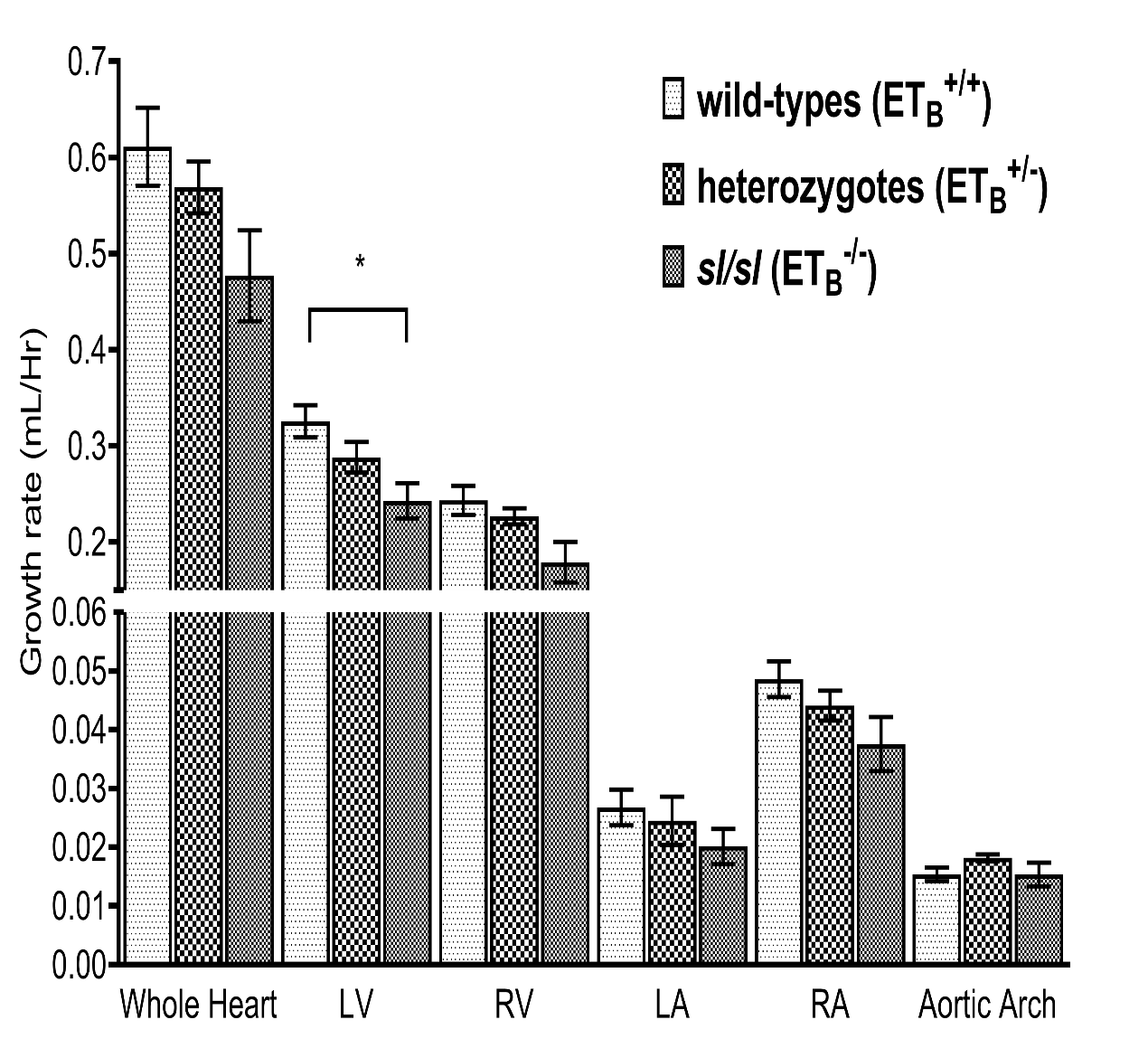

Supplement: Supplementary file 2 — Additional file 2: Figure 2. Stepwise growth rate reduction was associated with decreasing functional ETB. Stepwise decrease in cardiac growth rates corresponded to reducing ETB copies. As shown, wild-type has highest growth rates, followed by heterozygotes in the middle, and lowest in sl/sl rat: whole heart (0.61 mm3/Hr; 0.57 mm3/Hr; 0.48 mm3/Hr), LV(0.33 mm3/Hr; 0.29 mm3/Hr; 0.24 mm3/Hr), RV (0.24 mm3/Hr; 0.23 mm3/Hr; 0.18 mm3/Hr), LA (0.027 mm3/Hr; 0.024 mm3/Hr; 0.020 mm3/Hr), and RA (0.049 mm3/Hr; 0.044 mm3/Hr; 0.038 mm3/Hr). Wild-type and heterozygotes have relatively small growth rate variations when comparing their respective differences to sl/sl rat. No consistent correlations with genotype can be deduced from aortic arch measurements: wild-type (0.015 mm3/Hr), heterozygotes (0.018 mm3/Hr), and sl/sl (0.015 mm3/Hr). RA = Right Atrium; RV = Right Ventricle; LA = Left Atrium; LV = Left Ventricle. *: statistically significant in comparison to ETB−/− group, p ≤ 0.05. [file 12872_2021_2281_MOESM2_ESM.tif]

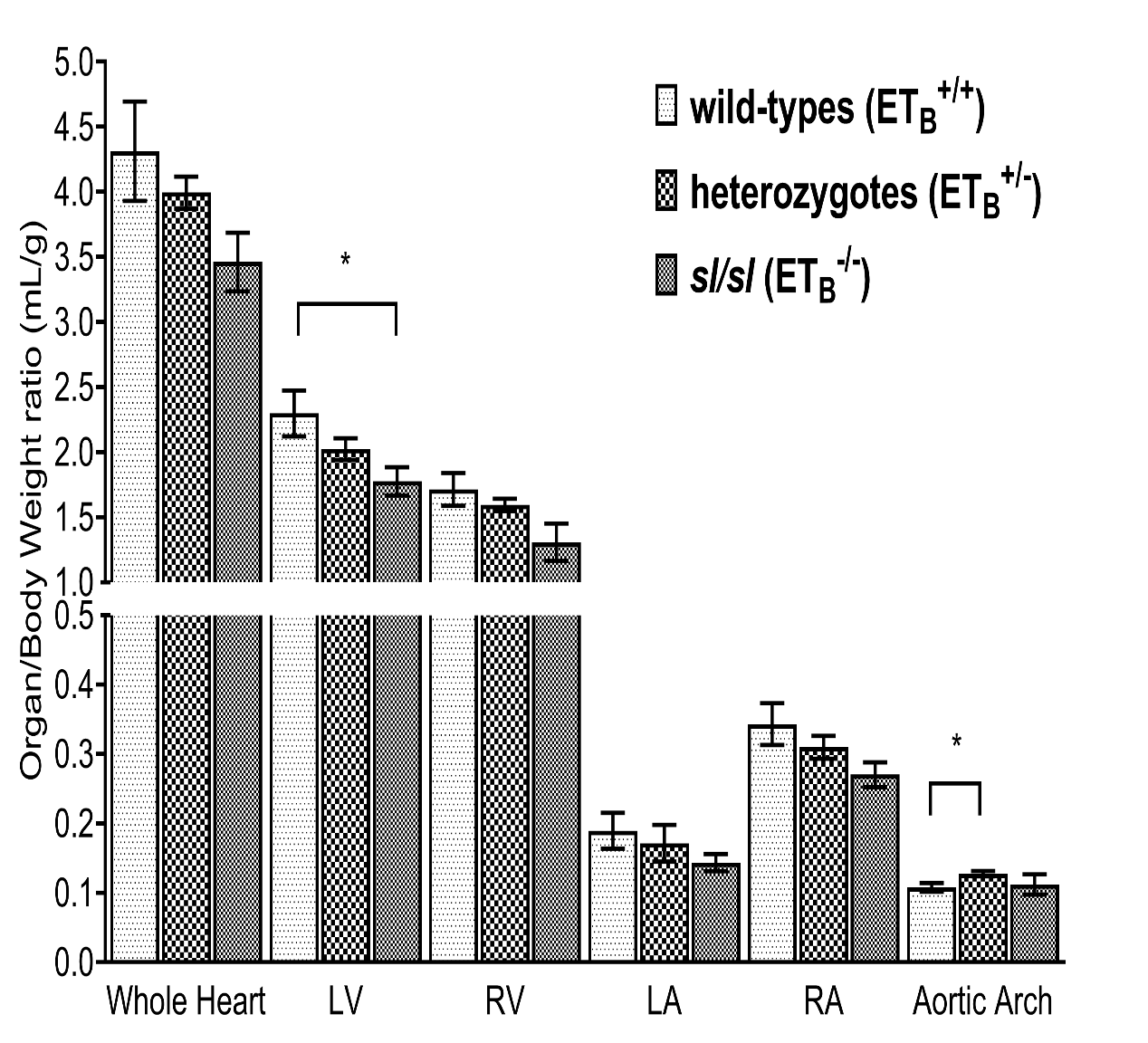

Supplement: Supplementary file 3 — Additional file 3: Figure 3. stepwise reduction in organ/bodyweight ratio associated with decreasing copies of functional ETB. Positive correlations between cardiac organ/bodyweight indices and ETB gene copy was demonstrated. Wild-type has the highest ratio, followed by heterozygotes in the middle, and sl/sl having the lowest: whole heart (4.31 mm3/g; 4.00 mm3/g; 3.46 mm3/g), LV(2.30 mm3/g; 2.02 mm3/g; 1.77 mm3/g), RV (1.71 mm3/g; 1.59 mm3/g; 1.31 mm3/g), LA (0.19 mm3/g; 0.17 mm3/g; 0.14 mm3/g), and RA (0.34 mm3/g; 0.31 mm3/g; 0.27 mm3/g). Minor difference was presented between wild-type and heterozygote when comparing to the reductions observed in sl/sl rats. In conjunction with Figure 1, this supported ETB mutation has intrinsic effect on cardiac development. No consistent trend associating with genotype can be deduced from aortic arch measurements: wild-type (0.11 mm3/g), heterozygotes (0.13 mm3/g), and sl/sl (0.11 mm3/g). RA = Right Atrium; RV = Right Ventricle; LA = Left Atrium; LV = Left Ventricle. *: statistically significant in comparison to ETB−/− group, p ≤ 0.05. [file 12872_2021_2281_MOESM3_ESM.tif]
