# Supplementary material for: Structural heart defects associated with ETB mutation, a cause of Hirschsprung disease
Source: BMC Cardiovasc Disord. 2021 Oct 2;21:475. doi: 10.1186/s12872-021-02281-2 (PMC8487587; doi:10.1186/s12872-021-02281-2)
Supplement: Supplementary file 4 — Additional file 4. Supplementary Table 1: sl/sl rat has reduced luminal width of aortic arch when comparing to the control groups. Two-dimensional measurement at the entry of aortic valve showed sl/sl rat having smaller intravascular width comparing to the control group. This trend persisted when respective age and bodyweight were standardized, as shown by the comparison of width growth-rate and width/bodyweight ratio, suggesting possible vasoconstriction. [file 12872_2021_2281_MOESM4_ESM.docx]

| Dimensions | *sl/sl*  (ET_B_^-/-^) | Control  (ET_B_^+/+^ & ET_B_^+/-^) | Wild-types  (ET_B_^+/+^) | Heterozygotes  (ET_B_^+/-^) |
| --- | --- | --- | --- | --- |
| Aortic Arch (AA) | | | | |
| AA luminal width (mm) | 0.43 | 0.59 | 0.53 | 0.65 |
| AA luminal width growth-rate (mm/Hr) | 0.0052 | 0.0063 | 0.0058 | 0.0068 |
| AA luminal width/bodyweight (mm/g) | 0.038 | 0.044 | 0.041 | 0.048 |

Supplementary Table 1: *sl/sl* rat has reduced luminal width of aortic arch when comparing to the control groups.

Two-dimensional measurement at the entry of aortic valve showed *sl/sl* rat having smaller intravascular width comparing to the control group. This trend persisted when respective age and bodyweight were standardized, as shown by the comparison of width growth-rate and width/bodyweight ratio, suggesting possible vasoconstriction.
